# Optimized Single-Step Recovery of Lipophilic and Hydrophilic Compounds from Raspberry, Strawberry and Blackberry Pomaces Using a Simultaneous Ultrasound-Enzyme-Assisted Extraction (UEAE)

**DOI:** 10.3390/antiox12101793

**Published:** 2023-09-22

**Authors:** Morag Davidson, François Louvet, Emmanuelle Meudec, Cornelia Landolt, Karine Grenier, Sandrine Périno, Tan-Sothéa Ouk, Naïma Saad

**Affiliations:** 1 Univ. Limoges, LABCiS, UR 22722, F-87000 Limoges, France; morag.davidson@etu.unilim.fr (M.D.); cornelia.landolt@unilim.fr (C.L.); karine.grenier@unilim.fr (K.G.); tan-sothea.ouk@unilim.fr (T.-S.O.); 2 ENSIL-ENSCI Formation: Céramique Industrielle, ESTER, Université de Limoges, 87068 Limoges, France; francois.louvet@unilim.fr; 3 SPO, INRAE, Institut Agro, Université de Montpellier, 34060 Montpellier, France; emmanuelle.meudec@inrae.fr; 4 INRAE, PROBE Research Infrastructure, Polyphenol Analytical Facility, 34060 Montpellier, France; 5 Équipe GREEN, UMR 408 SQPOV, Avignon Université, F-84000 Avignon, France; sandrine.perino@univ-avignon.fr

**Keywords:** berry pomaces, alkaline protease, ultrasound-enzyme-assisted extraction, DSD optimization, polyphenols, antioxidant activity, oil

## Abstract

An ultrasound-enzyme-assisted extraction (UEAE) was optimized to extract, simultaneously, the hydrophilic and lipophilic compounds from three berry pomaces (raspberry, strawberry and blackberry). First, an enzyme screening designated a thermostable alkaline protease as the most suitable enzyme to recover, in an aqueous medium, the highest yields of polyphenols and oil in the most efficient way. Secondly, the selected enzyme was coupled to ultrasounds (US) in sequential and simultaneous combinations. The simultaneous US–alkaline enzyme combination was selected as a one-single-step process and was then optimized by definitive screening design (DSD). The optimized parameters were: US amplitude, 20% (raspberry pomace) or 70% (strawberry and blackberry pomaces); pH, 8; E/S ratio, 1% (*w*/*w*); S/L ratio, 6% (*w*/*v*); extraction time, 30 min; temperature, 60 °C. Compared to conventional extractions using organic solvents, the UEAE extracted all the polyphenols, with around 75% of the active polyphenols (measured by the DPPH^●^ method) and up to 75% of the initial oil from the berry pomaces. Characterized lipophilic compounds were rich in polyunsaturated fatty acids (PUFAs), tocols and phytosterols. The polyphenolics were analyzed by UPLC-MS/MS; characteristic ellagitannins of the *Rosaceae* family (sanguiin H-6 or agrimoniin, sanguiin H-10, …) and ellagic acid conjugates were found as the major components.

## 1. Introduction

For 10 years, 100 million tons of berries have been produced globally each year (http://www.fao.org/faostat/en/#data, accessed on 1 February 2022). After the first industrial transformation, 20% to 30% (*w*/*w*) of the fruits are discarded [1], corresponding to the peels, the seeds and the stems, called “pomace”. These wastes contain bioactive compounds, either hydrophilic (polyphenols, sugars, proteins) or lipophilic (unsaturated fatty acids, phytosterols, tocols). Berry pomace polyphenolic compounds comprise phenolic acids, flavonoids and anthocyanins, but mostly ellagitannins (ETs) [2]. Raspberry, blackberry and strawberry seeds especially present a high content of ETs, which are monomers and oligomers of hydrolyzed tannins [3]. These compounds are ellagic acid conjugates and are characterized by their special structure of ester bond between the hexahydroxydiphenyl acid (HHDP) molecule and the saccharide conjugate, most commonly of glucose [4].

Berry seed oils contain great amounts of unsaturated fatty acids, which represent at least three-quarters of the total fatty acid content, with an omega-6/omega-3 ratio lower than 5, synonymous with health benefits [5,6]. They are also rich in phytosterols (mainly β-sitosterol) and tocols (α-, γ-, δ-tocopherols and γ-tocotrienol, mostly) [5]. These compounds are known for their multiple benefits on human health. Unsaturated fatty acids are usually associated with a reduced risk of cardiovascular diseases [7], tocols fight against molecular aging [8] and phytosterols can reduce the quantity of cholesterol in blood [9]. Polyphenols are mainly known for their antioxidant, anticancer, antimicrobial and anti-inflammatory properties [10]. Due to these contents in bioactive compounds, berry pomaces could have more applications than being used as animal feed or fertilizer. Several works have indeed demonstrated in vitro and in vivo biological activities displayed by extracts obtained from berry pomaces (antioxidant [11], anticancer [12], antiviral [13] and more), with promising applications for food, cosmetic and pharmaceutical industries.

The development of eco-friendly extraction methods of natural products is a hot research topic in chemistry and technology. Green extraction processes represent alternatives to conventional maceration and organic solvent extraction methods. Several eco-extraction methods are already used to extract bioactive compounds from berry pomaces, such as enzyme-assisted extractions (EAE) [14], microwave-assisted extractions [15], pulsed electric field-assisted extractions [16], pressurized-liquid extractions [12], supercritical fluid extractions [17] or ultrasound-assisted extractions (UAE) [11]. Simultaneous or sequential combinations of enzyme(s) with a physical treatment have also been explored as a way of intensifying extraction yields and/or overcoming the drawbacks of the enzyme technology when used alone (high solvent consumption, long extraction times and low recovery yields of lipophilic compounds). For example, Hu et al. (2019) extracted oil from cherry seeds by pre-treating them for 38 min with a combination of ultrasounds (US) and microwaves, before performing an EAE for 4 h with a cocktail of cellulase, hemicellulase and pectinase. Compared to a Soxhlet extraction, the oil extraction yields were similar, with comparable fatty acid profiles, but with a peroxide value 34% lower for the oil obtained with the green extraction [18]. The work of Xue et al. (2021) is an example of a simultaneous combination of a pectinase to US for the recovery of anthocyanins from raspberry wine residues in 30 min [19]. Nonetheless, to the best of our knowledge, no study was performed to recover simultaneously lipophilic and hydrophilic compounds from berry pomaces by coupling enzyme(s) to physical treatment(s), while it is known that a synergistic/additive effect exists between the biological activities of these two types of compounds [20].

Our previous work demonstrated that an alkaline protease was able to extract simultaneously polyphenols and oil from a raspberry pomace press-cake (PPC). In total, 148% of the total polyphenols, 125% of the active polyphenols and 38% of the oil were recovered in 2 h in an aqueous medium (compared to organic solvent extractions conducted in comparison) [14]. The possible highest oil yield, coupled with the lowest possible extraction costs, could be achieved with an intensification. Our choice fell on UAE, as this extraction method has gained a lot of interest nowadays, being low-cost, efficient and easy to implement [21]. The alternation of compression and rarefaction cycles leads to the formation and the growth of cavitation bubbles, formed from dissolved gases present in the solvent. When they reach their critical size, the bubbles explode, generating a hot spot and creating microjets and shockwaves that degrade the vegetal matrix [21]. Additionally, combining enzymes with US could improve the enzyme activity by modifying its structural conformation [22].

This present work aims to combine simultaneously enzyme(s) to a US treatment to increase the extraction yields of both hydrophilic and lipophilic compounds, in an aqueous medium, from three berry pomaces (raspberry, strawberry and blackberry). Four steps were followed: (i) a characterization of the pomaces; (ii) an enzyme screening to choose the most suitable enzyme(s) between a preselection; (iii) a screening to choose the best combination of enzyme(s) to US; (iv) a final optimization of the process by definitive screening design (DSD). The optimized extracts were fully characterized.

## 2. Material and Methods

### 2.1. Chemicals and Reagents

Hexane, heptane, acetonitrile, methanol, ethanol and acetone were purchased from VWR BDH Chemicals (Fontenay-sous-Bois, France). Pyridine, BSTFA + 1% TMSC, DPPH, Folin–Ciocalteu, sodium carbonate, ellagic acid, gallic acid, fatty acids, phytosterol standards, α-cholestane and enzymes were obtained from Sigma-Aldrich (Saint Quentin-Fallavier, France). Purchased enzymes were a cocktail of arabanase, cellulase, β-glucanase, hemicellulase and xylanase from *Aspergillus aculeatus* (Viscozyme L, ≥100 FBGU/g), acid protease from *Rhizopus* sp. (P0107, ≥0.2 U/mg) and an alkaline protease from *Bacillus licheniformis* (Alcalase 2.4 L FG, ≥2.4 U/g). Tocotrienol and tocopherol mixed solution standard came from ChromaDex (Irvine, CA, USA). All other chemicals and reagents were of analytical grade and purchased from Sigma-Aldrich (Saint Quentin-Fallavier, France).

### 2.2. Pomaces Preparation

Raspberry, strawberry and blackberry frozen pomaces were kindly provided by Andros, (Biars-sur-Céré, France). The raspberry pomace was constituted almost entirely of seeds (>95% *w*/*w*), while the strawberry and blackberry pomaces were made up of a mix of seeds and peels. They were oven-dried at 40 °C for 48 h and stored in a closed container at 4 °C in the dark. The dried pomaces (DP) were milled using a cutting mill SM 300 (Retsch) and sieved with a vibratory sieve shaker AS 200 equipped with a 250 µm sieve (Retsch, Verder, Eragny-sur-Oise, France).

### 2.3. Determination of the Pomace Proximate Compositions

#### 2.3.1. Polyphenol Content

Polyphenols were extracted from the pomace following a method described by Ayoub et al. (2016) [23]. In total, 10 g of milled DP was mixed with 125 mL of methanol:acetone:water mixture (7:7:6, *v*/*v*/*v*). The medium was sonicated for 20 min in an ultrasound bath at room temperature (RT). The supernatant was collected after centrifugation (15 min at 3500 rpm). The resulting residue was then extracted twice more following the same procedure. The organic phases were pooled and the methanol and acetone were evaporated at 40 °C using a rotary evaporator under a vacuum (Hei-vap-Advantage, Heidolph, Schwabach, Germany). The final aqueous extract was lyophilized, and the dried extract was stored at −20 °C.

#### 2.3.2. Oil Extraction

Raspberry, strawberry and blackberry pomace oils were extracted by exhaustion with hexane. A total of 20 g of milled DP was mixed with 200 mL of hexane. The extraction was activated with a 10 min passage in an ultrasonic bath and the mixture was then stirred for 1 h at RT. The organic phase was separated by centrifugation (15 min at 3500 rpm) and the residue was extracted once more as previously described. Hexane fractions were pooled and evaporated at 40 °C using a Heidolph rotary evaporator under a vacuum (Hei-vap-Advantage, Heidolph, Schwabach, Germany), and oils were stored at 4 °C under nitrogen.

#### 2.3.3. Protein and Ashes Contents

Kjeldahl method (N × 6.25) was used to quantify the protein content on 1 g of each milled DP. 2006 Digestor and 2100 Kjeltec distillation units (Foss Tecator, Höganäs, Sweden) were used.

Ash content of the three pomaces was determined after total mineralization of 1 g of each DP for 5 h at 600 °C.

#### 2.3.4. Fiber Content Determination

The dietary fiber (DF) contents of each berry pomace were investigated. DFs are classified regarding their solubility in water, pectin and some hemicellulose are soluble, while cellulose and lignin are insoluble. Before analysis, the three DP were defatted using hexane as described in Section 2.3.2. The defatted pomace residues were left overnight at RT (elimination of residual hexane solvent), and then put 1 h at 40 °C. The soluble starch, sugars and phenolic compounds were removed by boiling 20 g of defatted pomace in 200 mL of water for 10 min. The supernatant was separated by centrifugation (15 min at 3500 rpm) and the blanched-defatted pomace residues were dried for 48 h at 60 °C [24].

Firstly, the total dietary fiber content was quantified using the AACC methods 32-05.01 and the AOAC method 985.29 (Megazyme, Wicklow, Ireland) and their soluble and insoluble parts were estimated according to the AOCC method 991.43 and AACC method 32.07.01 (Megazyme, Wicklow, Ireland).

Secondly, a sequential fractionation of the fibers (pectin, hemicellulose, cellulose and lignin) was made up according to Alba et al. (2018) [25] and Bunzel et al. (2011) [26]. Pectin, lignin, hemicellulose and cellulose yields were determined gravimetrically following the fractionation protocol of Alba et al. (2018) [25].

For their part, Klason lignin contents were determined according to Bunzel et al. (2011) [26]. One g of total DFs was treated with 20 mL of 12 M H_2_SO_4_, in an ice bath, for 2 h under continuous agitation. A total of 130 mL of water was then added, and the mixture was heated in a boiling water bath for 2 h. The insoluble residue was filtered out of the mixture, washed with 250 mL of water and 50 mL of acetone and dried overnight at 60 °C. The residue was weighed and then mineralized for 5 h at 600 °C. Klason lignin contents were calculated by subtracting the ashes’ weight from the residue weight.

#### 2.3.5. Polyphenol Analyses

Prior to any analysis, lyophilized polyphenolic extracts were dissolved in ethanol 50% solution (*v*/*v*) at an appropriate concentration and their total phenolic content (TPC) was determined thanks to the Singleton and Rossi (1965) method [27]. Briefly, 200 µL of the extract was mixed with 1 mL of 1:10 aqueously diluted Folin–Ciocalteu reagent. After 5 min of incubation in the dark at RT, 800 µL of Na_2_CO_3_ solution (75 g/L in water) was added. The mixture was left in the dark at RT for 2 h. The absorbance was measured at 765 nm. Contents were calculated using a calibration curve established with gallic acid as standard (12.5 to 200 mg/L in water). Results are expressed in g of gallic acid equivalents (GAE) per 100 g of milled DP.

Anthocyanin content was assessed by pH differential method [28]. In total, 200 µL of appropriate diluted extract was mixed with 800 µL of KCl 0.025 M solution (pH 1.0) or with CH_3_COONa 0.4 M solution (pH 4.5). The mixtures were left 15 min in the dark at RT and absorbances were read successively at 520 and 700 nm. The anthocyanin absorbance was calculated as follows:

$$A={\left(A_{520\mathrm{nm}}-{\;A}_{700\mathrm{nm}}\right)}_{\mathrm{pH}\;1.0}-{\left(A_{520\mathrm{nm}}-{\;A}_{700\mathrm{nm}}\right)}_{\mathrm{pH}\;4.5}$$

Anthocyanin contents were estimated using a calibration curve established with cyanidin-3-glucoside as standard (2 to 20 mg/L in water).

Free ellagic acid and ET contents were assessed according to Bobinaitė et al. (2013) [29]. Free ellagic acids present in the extract were analyzed without acid hydrolysis, whereas Ets were determined after HCl hydrolysis in methanol and were estimated as total ellagic acid equivalent. Final ET content was quantified by subtracting the free ellagic acid amount from the total ellagic acid determined after acid hydrolysis. In both cases, analyses were carried out in an HPLC system (Shimadzu, Kyoto, Japan) equipped with an LC-20A pump and an SPD M20A diode array detector. Separation was performed on a Gemini-NX C18 RP column (250 × 4.60 mm, 5 µm, Phenomenex, Torrance, CA, USA). The column was maintained at 30 °C and eluted with a mobile phase composed of 1% formic acid in water (eluent A) and acetonitrile:methanol (85:15, *v*/*v*) (eluent B). Elution was performed in gradient mode in accordance with Bobinaitė et al. (2013) [29]. Ellagic acid was detected and quantified at 367 nm thanks to a calibration curve established with ellagic acid (from 0.075 to 0.5 mg/mL in methanol 95% solution (*v*/*v*)). The results are expressed as ellagic acid equivalents per 100 g of milled DP.

#### 2.3.6. Antioxidant Activity

Extracts’ antioxidant activity was assessed according to Brand-Williams et al.’s (1995) method [30]. Briefly, 0.5 mL of either extract solution or absolute ethanol was mixed with 1 mL of 0.2 mM DPPH solution in ethanol. The reaction mixtures were shaken vigorously and left in the dark at room temperature for 30 min. Absorbance was measured at 517 nm. DPPH^●^ radical-scavenging activity was expressed according to the following equation:

$$\mathrm{Antioxidant}\;\mathrm{activity}\;\left(\%\right)=\left(1-\frac{A_{\mathrm{sample}}-A_{\mathrm{control}}}{A_{\mathrm{blank}}}\right)\times 100$$

where A_sample_ is the absorbance of the extract with DPPH, A_control_ is the absorbance of extract in ethanol (without DPPH) and A_blank_ is the absorbance of DPPH in ethanol. Measurements were performed in triplicate. A calibration curve was established using gallic acid as a standard in the range from 2 to 10 mg/L. The results were expressed as g of GAE recovered from 100 g milled DP.

#### 2.3.7. Oil Content Characterization

The fatty acid composition of the extracted oils was determined by GC/MS as fatty acid methyl esters (FAMEs). A total of 50 mg of each pomace oil were mixed with 5 mg of C17:0 used as an internal standard. The samples were first saponified with 0.5 M KOH in methanol and then methylated in the presence of 14% BF_3_/MeOH. After n-heptane extraction, the FAMEs were analyzed with a QP 2010 SE GC/MS system (Shimadzu Corporation, Kyoto, Japan). A BPX70 capillary column (30 m × 0.25 mm ID, 0.25 µm, SGE Analytical Science, Melbourne, Australia) was used in isothermal mode at 180 °C with helium as a carrier gas at 1.0 mL/min. A total of 1 µL of the FAMEs was injected into the column using a 100:1 split ratio. FAMEs were identified by matching their mass spectra and retention times with those of a standard mixture and quantified by determining their relative peak area.

The α-, β-, γ-, and δ-tocopherol and tocotrienol contents were determined according to Pieszka et al. (2015) [31] using a normal-phase HPLC. Analyses were performed on a Shimadzu HPLC system equipped with a Nucleodure 100-5 normal phase column (250 × 4.6 mm, Macherey-Nagel, Düren, Germany). The system was operated in isocratic mode at a flow rate of 1.6 mL/min with hexane:ethyl acetate:acetic acid (97.3:1.8:0.9, *v*/*v*/*v*) as a mobile phase. The separations were carried out at 30 °C and the compounds were UV detected at 292 nm. Identification and quantification were performed using standard solutions containing a mixture of tocopherols and tocotrienols from ChromaDex (Irvine, CA, USA).

Phytosterol contents were determined according to Pieszka et al.’s (2015) method [31]. A-cholestan was used as an internal standard. After a saponification step, a silylation of the extracted phytosterols was undertaken with BSTFA reagent. The derivatized sterols were then analyzed on a QP 2010 SE GC/MS equipped with a ZB 5-HT capillary column (30 m × 0.25 mm ID, 0.25 μm, Phenomenex (Torrance, CA, USA)). Helium was used as carrier gas at a flow rate of 1.0 mL/min. The following temperature program was used: 60 °C for 1 min and then 8 °C/min ramp to 340 °C, held for 10 min at this temperature. Injector and detector temperatures were set at a temperature of 300 and 260 °C, respectively. In total, 1 µL sample was injected using a 20:1 split ratio. Identification and quantification were performed using a mixture of standards.

### 2.4. Simultaneous Ultrasound-Enzyme-Assisted Extraction (UEAE) Process Development

This study focuses on the simultaneous recovery of lipidic and phenolic compounds from three berry pomaces (raspberry, strawberry and blackberry). The extraction procedure was divided into three parts: (i) an enzyme-assisted extraction, (ii) an ultrasound-enzyme-assisted extraction and (iii) an optimization of the selected process by DSD. The efficiency of each extraction procedure for the simultaneous recovery of polyphenols and oil was estimated by comparing their rates to those obtained with organic solvent extractions (see Section 2.3.1 and Section 2.3.2).

#### 2.4.1. Enzyme-Assisted Extraction (EAE)

One mix of carbohydrolases (Viscozyme L) and two proteases, one acid protease (P0107) and one thermostable alkaline protease (Alcalase 2.4 L FG), were evaluated alone or combined (sequentially or simultaneously). A total of 10 g of each milled DP (≤250 µm) and 200 mL of Milli-Q water with a solid/liquid (S/L) ratio of 5% (*w*/*v*) were placed in a 500 mL double glass wall reactor (Mac Technologie, Fontenay Tresigny, France). Temperature was regulated by a cryostat (RW-0540G, Lab. Companion, UK) and pH was adjusted using 1 M NaOH or 1 M HCl. The medium was homogenized with mechanical stirring (Mac Technologie, Fontenay Tresigny, France). The enzyme-to-solid (E/S) ratio was fixed at 2% (*w*/*w*). pH and temperature were chosen according to the enzyme optimum parameters specified by the suppliers. The extraction conditions were fixed: (i) for the carbohydrolases, pH 4 at 45 °C for 2 h; (ii) for the acid protease, pH 4; (iii) for the alkaline protease, pH 8, and temperature was set at 50 °C for 1 h for both proteases. Following the extractions, the supernatants (oil in water emulsions) were carefully recovered from the residues by centrifugation (15 min at 3500 rpm). The total polyphenol and oil rates, as well as the polyphenol antioxidant activity, were determined as described above (Section 2.3.1, Section 2.3.2 and Section 2.3.6). Control extractions were made in the same extraction conditions without enzyme(s). Conventional extractions using organic solvents (see Section 2.3) were conducted on the 250 µm milled pomaces to determine the reference yields of polyphenols, antioxidant activity and oil. The yields of extraction were calculated as g per 100 g of milled DP and the results were expressed in percentage of efficiency compared to those obtained by conventional extractions.

#### 2.4.2. Ultrasound-Enzyme-Assisted Extraction (UEAE)

The most suitable enzyme(s) system selected above (Section 2.4.1) was used for the UEAE. Five extraction conditions were compared: (i) enzyme alone (EAE), (ii) US alone (UAE), (iii) sequential US pre-treatment followed by enzyme extraction (UAE → EAE) or (iv) enzyme pre-treatment followed by US extraction (EAE → UAE) and finally (v) simultaneous enzyme-US extraction (UEAE). A control extraction without enzyme and US treatment was also carried out. All extractions were realized in a 650 mL double-mentor reactor (Legallais, Caen, France). For all tests, the temperature was set at 50 °C using a cryostat (Huber, Berching, Germany), pH 8, pomace particle size ≤ 250 µm and S/L ratio = 5% (*w*/*v* in water). Extraction time was set at 1 h for the EAE, UAE, UEAE and the control. For the sequential tests, pre-treatment durations were 30 min followed by 30 min of extraction. For extractions using the enzyme(s), the E/S ratio was fixed at 2% (*w*/*w*) and the medium was stirred with an overhead stirrer (OST basic, IKA, Staufen, Germany). Ultrasound-assisted extractions were performed with a 20 mm diameter ultrasonic probe (1 kW, UIP, 1000 hdt, Hielscher Ultrasonics GmbH, Teltow, Germany) at 70% amplitude. Following extractions, supernatants were separated from residues by centrifugation (15 min at 3500 rpm) and the total polyphenols, antioxidant activity and oil rates were assessed according to Section 2.3.1, Section 2.3.2 and Section 2.3.6. The yields of extraction were calculated as g per 100 g of milled DP and the results were expressed in percentage of efficiency compared to those obtained by conventional extractions.

#### 2.4.3. UEAE Optimization by Experimental Design

A definitive screening design (DSD) [32] was applied to determine the best combination of UEAE variables to optimize simultaneously the recoveries of hydrophilic and lipophilic compounds from the raspberry, strawberry and blackberry pomaces. First, raspberry pomace was used as a model for the study, then the optimized and validated parameters were applied to the two other pomaces.

Six independent factors were considered as key factors: US amplitude (%), pH, E/S ratio (%, *w*/*w*), S/L ratio (%, *w*/*v*), time (min) and temperature (°C). Three levels were required per factor (minimum, centered, maximum). The number of experiments was defined by: *N* = 2 *k* + 1, where *k* represents the number of independent factors, which leads to 13 experiments, realized in triplicate. Three responses were considered: the total polyphenol content, antioxidant activity and oil extraction yields (assessed according to Section 2.3.1, Section 2.3.2 and Section 2.3.6). The yields of extraction were calculated as g per 100 g of milled DP.

To evaluate the results, a loss function was used. This tool allows one to give one score to an experiment, by assessing the yields and the corresponding deviations. A loss function tending to 0% is synonymous with the best extraction parameters, with the highest yields and the lowest standard deviations, while a loss function of 100% corresponds to the worst experiment.

### 2.5. UPLC-DAD-ESI-MS/MS Polyphenols Analysis

The extracts were analyzed by UHPLC (Vanquish, Thermo Scientific, Waltham, MA, USA) using an Acquity UPLC HSST3 C18 column (100 mm × 1 mm ID, 1.7 µm; Waters, Milford, MA, USA). The mobile phase consisted of (A) water:formic acid (99:1, *v*/*v*) and (B) acetonitrile:water:formic acid (79.5:19.5:1, *v*/*v*/*v*).

Flow rate was 0.22 mL/min. The elution program was as follows: isocratic for 1.5 min with 2% B, 2–12% B (1.5–4.5 min), isocratic with 12% B (4.5–7 min), 12–24% B (7–12 min), 24–48% B (12–15 min), 48–60% B (15–16 min), 60–100% B (16–17 min). The column and the injector temperatures were maintained at 35 and 10 °C, respectively. The injection volume was 0.5 µL.

The UHPLC system was coupled with a DAD covering the full range of acquisition (190–600 nm) and an HRMS (Orbitrap ExplorisTM 480, Thermo Scientific, Waltham, MA, USA) equipped with an electrospray ionization probe. The HRMS was operated in negative ion mode. The parameters for the ion source were as follows: ion transfer tube temperature: 280 °C; voltage: 2500 V; sheat gas: 40 a.u.; auxiliary gas: 10 a.u.; sweep gas: 2 a.u.; vaporizer temperature: 300 °C; mass range: 100–1800 Daltons (Da); resolution: 240,000 and 480,000. XcaliburTM 4.4 (Thermo Scientific, Waltham, MA, USA) was used for instrument control, data acquisition and data analysis.

The tentative identification was based on mass spectrum, accurate mass, characteristic fragmentation, UV spectrum and time retention available in the literature.

### 2.6. Statistics

Statistical analyses were performed using XLSTAT (16.63 version) using one-way analyses of variance (ANOVA). Each assay was conducted and analyzed in triplicate. Data are presented as mean value ± standard deviation.

## 3. Results and Discussion

### 3.1. Pomaces’ Chemical Compositions

The proximate chemical compositions of the raspberry, strawberry and blackberry pomaces are given in Table 1. The raspberry pomace was constituted of both hydrophilic and lipidic compounds. The main hydrophilic compounds were fibers, at a level of 80 ± 3 g/100 g of DP. Insoluble fibers constituted most of the fiber contents (>90% of the total fibers), with cellulose identified as the prevalent one (79 ± 2% of the total fiber content), followed by Klason lignin (34 ± 0.4%). Hemicellulose weighed 5.9 ± 0.3% of the total fiber content. Proteins were quantified at 8 ± 0.4 g/100 g DP, moisture at 4.7 g/100 g DP and ashes were found in a minor proportion (1.4 ± 0.07 g/100 g DP). TPC was evaluated at 4.7 ± 0.2 g/100 g DP. These results are similar to the ones reported in previous works on raspberry pomace [14,33]. The proximate compositions of the strawberry and blackberry pomaces, presented in Table 1, were close to the one obtained for the raspberry pomace, except for the strawberry pomace oil content, which was twice as low (6.3 ± 0.04 g/100 g DP), and the blackberry pomace fiber content, which was 20% lower (62 ± 2 g/100 g DP). Higher contents in lipids (12.03 ± 0.4 g/100 g DP) were previously reported for a strawberry pomace, which is linked to the quantity of seeds in the pomace, but other contents were similar [12]. Almost identical results were reported for a blackberry pomace press-cake (PPC), except for the lipidic content, which was 38% lower than our result (10.8 ± 0.3% of the PPC composition) [34].

According to the results illustrated in Supplementary Table S1, the oil extracted from the raspberry pomace by hexane was rich in polyunsaturated fatty acids (PUFAs), which accounted for 83 ± 11% of the total fatty acid content. The two dominant fatty acids were C18:2, n-6 (52 ± 7%) and C18:3, n-3 (32 ± 5%), with an omega-6/omega-3 ratio evaluated at 1.6 ± 0.2, matching the recommended ratio (1 to 5) associated to beneficial effects on human health [35]. This fatty acid profile was close to our previous results on raspberry PPC [14] and for a cold-pressed oil [33]. Moreover, a considerable amount of tocols was found (249 ± 8 mg/100 g of oil), γ-tocopherol being the most abundant one (76 ± 3% of the total tocol content). The oil was also rich in phytosterols, with 1164 ± 7 mg/100 g of oil, β-sitosterol being the most prevalent one (>95% of the total phytosterol content). A similar tocol content and profile were reported for a raspberry seed cold-pressed oil [5] and lower contents were found for a raspberry PPC, but with the same major components [14]. These differences can be due to variabilities in the fruits themselves (variety, species, geography, ripeness, growing conditions), in the oil extraction process and/or in the analytical methods used for quantification [36]. The oil extracted from the strawberry pomace showed the same fatty acid profile and similar phytosterol content and profile. Compared to the raspberry pomace tocol content, the strawberry pomace oil presented ~75% less tocols (66 ± 5 mg/100 g of oil), with twice as much *α*-tocopherol (38 ± 5% of the total tocol content). A similar fatty acid profile was reported by Van Hoed et al., 2009 [5]. However, the tocol and phytosterol contents were, respectively, 4 times higher and twice as low. Γ-tocopherol was mostly dominant (>90% of the total tocol content) and β-sitosterol was also found as the main phytosterol, but to a lesser extent (75% of the total phytosterol content) [5]. Blackberry pomace oil showed, globally, the same fatty acid profile as the raspberry pomace oil, except for the C18:3, n-3 content, which was twice as low (13 ± 2% of the total fatty acid content), leading to an omega-6/omega-3 ratio calculated at 4.2 ± 0.1. The tocol and phytosterol contents and their profiles were similar to those of Van Hoed et al., 2009 [5].

Regarding the polyphenolic fraction, the antioxidant activity, anthocyanin and ET contents were assessed. The antioxidant activity was calculated as the ratio between the active polyphenol content measured by the DPPH^●^ method and the TPC. The active polyphenol contents were assessed between 1.9 ± 0.04 and 2.9 ± 0.2 g/100 g of DP, leading to antioxidant activities assessed around 50% for the three pomaces. Otherwise, Ets were a major part of the measured phenolic compounds. Their contents represent 30 to 57% of the TPC, while blackberry pomace presents the highest value. These results concur with the literature, where Ets are stated to be the major phenolic compounds of berry seeds of the *Rosaceae* family [37]. Overall, anthocyanin contents in the three pomaces (<2% of the TPC) were significantly low, especially for raspberry anthocyanins, which were assessed at 10 ± 2 mg/100 g DP. Almost identical values were reported for a phenolic fraction obtained with an acetone solvent from defatted raspberry and strawberry pomaces [37]. Blackberry pomace phenolics displayed the highest anthocyanin content with a value of 99 ± 11 mg/100 g DP.

According to literature reports, phenolic compounds are generally attached to the plant constituents (proteins, carbohydrates) [38], while the oil is contained in cellular organelles, called oil bodies or oleosomes, which are enmeshed in a cytoplasmic network composed of proteins [39]. The use of hydrolytic enzymes, such as glycohydrolases and proteases, either alone or combined during the extraction process, can facilitate the dispersion of these lipophilic and hydrophilic compounds in an aqueous medium and result in an oil-in-water emulsion.

In light of the characterization results and the literature, one cocktail of carbohydrolases and two proteases will be tested for the first step of this study. Next, the most efficient enzyme(s) system will be coupled to a US treatment, for an optimal recovery of each berry pomace bioactive compound.

### 3.2. Enzyme Screening

**Figure 1:**
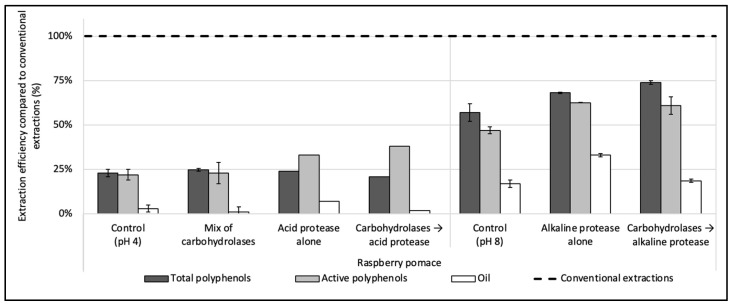
Enzyme screening for the simultaneous recovery of polyphenols and oil in water from raspberry pomace. A mix of carbohydrases (Viscozyme L), one acid protease (P0107) and one thermostable alkaline protease (Alcalase 2.4 L FG) were evaluated, alone or sequentially combined. The enzyme-to-solid ratio (E/S) was set at 2% (*w*/*w*) and extractions were conducted under the optimum conditions given by the suppliers. Control extractions were also realized with the same extraction parameters but without enzyme. The dashed line represents the yields obtained with conventional extractions taken as references (hexane for oil recovery and methanol:acetone:water (7:7:6, *v*/*v*/*v*) for total and active polyphenols recoveries).

Three enzymes were evaluated during the enzyme screening step: one cocktail of carbohydrases (Viscozyme L), one acid protease (P0107), and one thermostable alkaline protease (Alcalase 2.4 L FG), added either alone or sequentially. The results for the raspberry pomace can be found in Figure 1 and are expressed as a percentage of rate compared to organic solvent extractions used in the comparison (see Section 2.3.1, Section 2.3.2 and Section 2.3.6).

The treatments with the mix of carbohydrases and the acid protease, used either alone or combined, extracted hardly any of the oil contained in the raspberry pomace, with oil yields below 10% (<0.3 g of oil for 100 g DP) (Figure 1). Only the alkaline protease used alone had shown a good efficiency compared to the control without enzyme, with an oil recovery reaching 5.6 ± 0.2 (i.e., 32 ± 1% vs. 17 ± 2 of efficiency), while its combination with carbohydrases did not improve the oil extraction rate compared to the control realized without enzyme at pH 8. The oil is present in the seeds in the form of oil droplets, called oil bodies or oleosomes, enmeshed in a proteinic matrix. A layer constituted of phospholipids and proteins envelops the oil to protect it from physical and chemical stress. Due to the hydrophilic character of the proteins surrounding the oil and their isoelectric point, oil bodies can be extracted with an aqueous solvent [39] and by using alkaline proteases operating within an optimum pH range of pH 7–9. Their hydrolytic action on the proteins surrounding the oil thus allows one to release the oil in the aqueous solvent. Furthermore, the protease can also hydrolyze the cytoplasmic proteins, reducing its viscosity and facilitating the extraction of the oleosomes [40]. Conversely, the acid protease and the mix of carbohydrases were both used at pH 4. Due to the inability of the acid protease and the mix of carbohydrases to extract the oil from the pomace, these enzymes were discarded.

Regarding polyphenols, the results obtained by the combinations of the mix of carbohydrases/alkaline protease or by the alkaline protease used alone were similar and >10% higher than the control. Alone, the alkaline protease extracted 3.18 ± 0.02 g of the total polyphenols/100 g of DP (i.e., 68 ± 0.4% of efficiency) and 1.33 ± 0.01 g of the active polyphenols/100 g DP (63 ± 0.2%), while, when used after the mix of carbohydrases, the extraction efficiency of total polyphenols was slightly improved to 74 ± 1% without a significative change regarding active polyphenols.

The alkaline protease alone was thus chosen for further tests, as (i) it was the only enzyme to achieve a notable oil extraction recovery, (ii) it improved the total and active polyphenols recoveries compared to the control extraction and (iii) it is easy to implement, being a single-step extraction.

To summarize, the alkaline protease alone, with extraction conditions of particle size ≤ 250 µm; 50 °C; pH 8; enzyme concentration of 4.8 enzyme units/100 g of DP (E/S = 2% *w*/*w*); S/L ratio = 5% (*w*/*v*) and 1 h hydrolysis, recovered 3.2 ± 0.02 g of total polyphenols/100 g DP (68 ± 0.4% of efficiency); 1.33 ± 0.01 g of active polyphenols/100 g DP (63 ± 0.2%) and 5.1 ± 0.2 g of oil/100 g DP (32 ± 1%) from the raspberry pomace. In our previous work on raspberry PPC [14], under optimized extraction conditions (1.2 units of enzyme/100 g PPC; pH, 9; 60 °C and 2 h extraction time), the same enzyme extracted 38% of total PPC residual oil (2.2 ± 0.09 g/100 g PPC) while the total and active polyphenols recoveries were similar to current (i.e., 3.7 ± 0.04 g of total polyphenols and 1.46 ± 0.03 g of active polyphenols per 100 g PPC).

The oil yield achieved in this study stays significantly lower than the one obtained with the conventional extraction using hexane (5.1 ± 0.2 g of oil/100 g vs. 16 ± 0.3 g/100 g DP). An intensification of the extraction process with a physical treatment could improve the extraction rate of the oil. US being efficient and easy to implement, they were chosen to be coupled to the action of the alkaline protease. Moreover, Nadar and Rathod (2017) stated that US could have a beneficial impact on enzymes’ structural conformation, leading to higher extraction yields [22]. Promising results were obtained from Goula et al. (2016) on pomegranate seeds, where a pectinase was simultaneously coupled to US. Compared to the enzyme alone, the combination enhanced by 20% the oil extraction rate and reduced by 90% the extraction time (from 2 h to 10 min) [41].

Compared to the raspberry pomace, the strawberry and blackberry pomaces showed similar properties when treated with the same screened enzymes. The alkaline protease was the best enzyme model for the recovery of the highest yields of polyphenols and oil. For the next step of the study, the raspberry pomace was chosen as a model for the design of the combined enzyme-US extraction process and the optimized parameters will be subsequently transposed to the strawberry and blackberry pomaces.

### 3.3. UEAE Procedure

**Figure 2:**
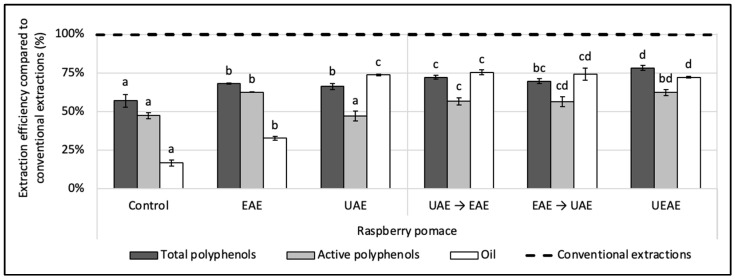
Effect of alkaline protease and US combinations on the simultaneous recovery of oil and polyphenols from raspberry pomace. A control extraction was carried out in the same extraction conditions without enzyme or US. Extractions with only the enzyme (EAE) and only US (UAE) were carried out as a comparison under the same conditions. Two sequential extractions were screened: a US pre-treatment followed by enzyme extraction and vice versa (EAE → UAE; UAE → EAE) as well as a simultaneous extraction with the enzyme used in situ with a US treatment (UEAE). Extraction parameters were fixed at: extraction time = 1 h or 30 min pre-treatment followed by 30 min extraction; US amplitude (if relevant) = 70%; pH = 8; E/S ratio (if relevant) = 2% (*w*/*w*); S/L ratio = 5% (*w*/*v*); T° = 50 °C; and size particle = < 250 µm. Alkaline protease from *Bacillus licheniformis* was used as the enzyme system (Alcalase 2.4 L FG, ≥2.4 U/g). The dashed line represents the yields obtained with conventional extractions (hexane for oil recovery and methanol:acetone:water (7:7:6, *v*/*v*/*v*) for total and active polyphenols recoveries). Mean values are expressed in efficiency (%) compared to conventional extractions with standard deviations (*n* = 3). Different letters indicate significant differences calculated by ANOVA (*p* < 0.05).

The alkaline protease was coupled sequentially and simultaneously with a US treatment for the simultaneous recovery of polyphenols and oil in an aqueous medium from the raspberry pomace. For the sequential procedures, two conditions were designed: (i) a US pre-treatment followed by an enzyme extraction (UAE → EAE) and (ii) an enzyme pre-treatment followed by a US extraction (EAE → UAE). For the simultaneous extraction procedure (UEAE), the enzyme was incorporated in situ into the US extraction reactor. Control extractions (i) with the enzyme alone (EAE), (ii) US alone (UAE) and (iii) without enzyme or US treatment (control extraction) were performed for comparison. The results presented in Figure 2 and are expressed in efficiency compared to conventional extractions realized with organic solvents. The extractions and the controls were performed under the same extraction parameters: extraction time (1 h for EAE, UAE, UEAE, and the control or 30 min of pre-treatment followed by 30 min of extraction for the sequential conditions); the temperature at 50 °C; pH, 8; S/L ratio, 5% (*w*/*v*); particle size, ≤250 µm; US amplitude (if relevant), 70%; and E/S ratio (if relevant), 2% (*w*/*w*).

US significantly improved the oil recovery compared to the enzyme alone, with the UAE reaching 75% of efficiency compared to 32% for the EAE (12 ± 0.09 g of oil/100 g vs. 5.6 ± 0.2 g/100 g DP). However, the UAE extracted 20% less active polyphenols than the EAE (1.00 ± 0.08 g/100 g vs. 1.33 ± 0.01 g/100 g DP). Such results were also observed by Varo et al. (2018) on phenolic compounds extracted from bilberry juice by-products, where the anthocyanin recovery was 21% lower after 1 h of UAE compared to the control extraction [42]. The cavitation phenomenon does not only damage the vegetal matrix, it also generates free radicals that can degrade the polyphenols. US is indeed an efficient way to improve the oil recovery, but in this case, they cannot be used alone, as they negatively impact the polyphenol activity.

Combining the action of the enzyme sequentially or simultaneously with US achieved the same oil extraction yield (73 ± 0.6% to 75 ± 2%) as the one obtained with the UAE (74 ± 0.6%). Concomitantly, the enzyme combinations with US improved the polyphenol extraction yields and their antioxidant activities. The total polyphenol yield was enhanced by 6% in sequential combinations and by 18% for UEAE (from 3.25 ± 0.08 to 3.65 ± 0.08 g/100 g DP). The active polyphenol content was enhanced in the same way, between 19% and 32%.

Since efficiencies obtained by the simultaneous UEAE were higher, this process was selected as the best combination for the next step of the study. This single-step extraction is also easier to implement. Additionally, the in situ use of an enzyme during US-assisted treatment is a conceptual break with the existing literature and shows an innovative character as it extracts simultaneously, in an aqueous medium, the lipophilic and hydrophilic bioactive compounds of the raspberry pomace.

### 3.4. UEAE Optimization by DSD

**Figure 3:**
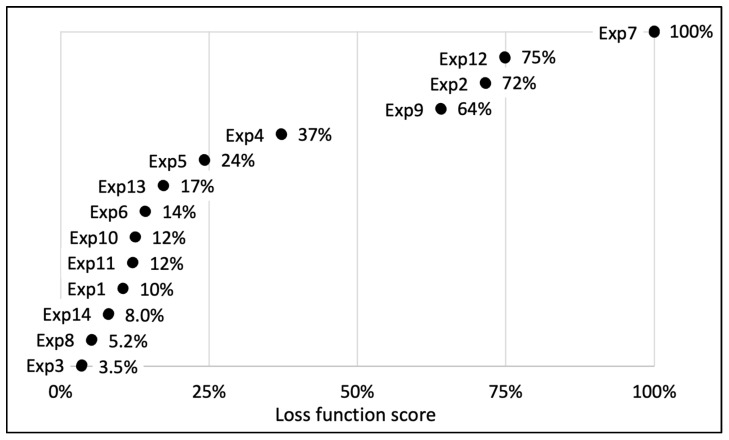
Representation of the distribution of the calculated loss functions for the 13 essays carried out during DSD experimental design for the optimization of UEAE procedure. Experiment 14 is a combination of experiments 3 and 8 and was designated as the optimized extraction. The represented loss function allows one to give one score for each experiment taking into account the obtained extraction yields (oil and polyphenols) and their corresponding deviations. A loss function tending to 0% is synonymous with the best extraction parameters, with the highest yields and the lowest standard deviations, while a loss function of 100% corresponds to the worst experiment.

A DSD was used to optimize the UEAE extraction procedure. Six parameters were selected: US amplitude, pH, E/S ratio, S/L ratio, extraction time and temperature. The DSD matrix, with the 6 parameters and their appropriate levels, is shown in Table 2, as well as the 13 conducted experiments. The goal of the optimization was twofold: maximizing the total polyphenol, active polyphenol and oil yields, while minimizing their standard deviations. The results can be found in Table 2, and their respective loss functions are represented in Figure 3.

Amongst the 13 experiments, experiments 3 and 8 showed the best extraction results on the raspberry pomace, with a loss function assessed, respectively, at 3.5% and 5.2% (Figure 3). Experiment 3 allowed us to extract 94 ± 4% of the total polyphenols (4.4 ± 0.2 g/100 g DP), 81 ± 3% of the active polyphenols (1.7 ± 0.05 g/100 g DP) and 84 ± 2% of the oil (13.2 ± 0.2 g/100 g DP) compared to conventional organic solvent extractions, while experiment 8 recovered 96 ± 3% of the total polyphenols (4.5 ± 0.1 g/100 g DP), 76 ± 3% of the active polyphenols (1.6 ± 0.06 g/100 g DP) and 78 ± 0.9% of the oil (12.3 ± 0.2 g/100 g DP). The extraction parameters of experiment 3 were fixed at: US amplitude = 70%; pH 8; E/S ratio = 3% (*w*/*w*); S/L ratio = 3% (*w*/*v*); extraction time = 30 min; 60 °C; while experiment 8 parameters were: US amplitude = 20%; pH 9; E/S ratio = 1% (*w*/*w*); S/L ratio = 6% (*w*/*v*); extraction time = 30 min; 60 °C.

To limit the US probe corrosion, optimize the extraction costs and secure the extract stability, experiment 14 was designed, based on the combination of experiments 3 and 8 parameters. Experiment 14 parameters were fixed: US amplitude = 20%; pH = 8; E/S ratio = 1% (*w*/*w*); S/L ratio = 6% (*w*/*v*); extraction time = 30 min; 60 °C. Its loss function was evaluated at 8.0%. This experiment and its parameters were designated as the optimized process, which allows the recovery of 4.9 ± 0.1 g of total polyphenols, 1.6 ± 0.02 g of active polyphenols and 12 ± 0.05 g of oil per 100 g of DP. These results represent, respectively, an efficiency of 104 ± 3%, 76 ± 0.5% and 76 ± 0.8% compared to organic solvent extractions (Table 2).

**Figure 4:**
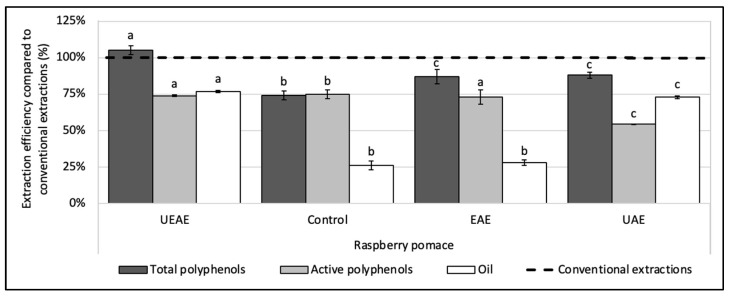
Synergistic effect of US and alkaline protease combination (UEAE) on the simultaneous recovery of lipophilic and hydrophilic compounds from raspberry pomace in an aqueous medium. Optimized extraction parameters were determined at: US amplitude = 70%; pH = 8; S/L ratio = 6% (*w*/*v*); E/S ratio = 1% (*w*/*w*); extraction time = 30 min; extraction temperature = 60 °C. Alkaline protease from *Bacillus licheniformis* (Alcalase 2.4 L FG, ≥2.4 U/g) was used as an enzyme system and particle size < 250 µm. Controls without enzyme or US (control extraction), with the enzyme alone (EAE) and with US alone (UAE) were carried out as a comparison under the same extraction parameters. The dashed line represents the yields obtained with conventional extractions (hexane for oil recovery and methanol:acetone:water (7:7:6, *v*/*v*/*v*) for total and active polyphenols recoveries). Mean values are expressed in efficiency (%) compared to conventional extractions with standard deviations (*n* = 3). Different letters indicate significant differences calculated by ANOVA (*p* < 0.05).

To assess the synergistic effect between the enzyme and US, two more experiments were realized with the optimized parameters, one with US alone (UAE) and one with the enzyme alone (EAE). Results in Figure 4 show that the EAE allowed for the recovery of almost all the polyphenols in their active form, but with a low oil rate, while the UAE boosted the oil extraction rate, but decreased the polyphenol activity as a side effect. The combination of the enzyme to US showed a real synergy, as the UEAE overcame in fine the two issues encountered with the enzyme and US used alone and allowed for the extraction of a high quantity of oil in an aqueous medium while preserving the polyphenol activity.

### 3.5. UEAE Optimized Parameters Transposition to Strawberry and Blackberry Pomaces

The optimized parameters obtained for the raspberry pomace were transposed to the strawberry and blackberry pomaces. At 20% US amplitude, the oil yields obtained from these two pomaces were lower than expected (). To overcome this issue, the US amplitude was increased to 70%. Other parameters were set at the same level as for the raspberry pomace.

The strawberry pomace followed the same case scenario as the raspberry pomace. The UEAE recovered the same quantity of oil as the UAE (3.5 ± 0.2 g/100 g DP), i.e., 56 ± 2% efficiency, and the same quantity of active polyphenols as the EAE. The final UEAE yields were 4.40 ± 0.09 g of total polyphenols, 1.6 ± 0.06 g of active polyphenols and 3.5 ± 0.2 g of oil per 100 g DP (respectively, 117 ± 3%, 84 ± 3% and 55 ± 3% of efficiency).

For the blackberry pomace, the quantities of active polyphenols extracted by the UEAE, the EAE and the UAE were similar (respectively, 78 ± 1%, 81 ± 2% and 71 ± 6%), but the UEAE showed an additional synergistic effect regarding the quantity of extracted oil with nearly 20% enhancement compared to the UAE (64 ± 0.6% vs. 47 ± 5%). The final UEAE yields were 5.3 ± 0.2 g of total polyphenols (105 ± 4% of efficiency), 2.20 ± 0.03 g of active polyphenols and 11 ± 0.1 g of oil per 100 g DP.

Overall, our three UEAE processes showed significant enhanced performances compared to conventional extractions (Table 3). They enable the extraction of all the polyphenolic compounds that have interesting antioxidant capacity (from 36% to 47% of the TPC). The slight diminution in the antioxidant activity compared to the conventional extractions may be explained by the extraction conditions applied during the UEAE process (60 °C and 20–70% US amplitude). The oil extraction rates for the UEAE processes are lower than the ones obtained with hexane (Table 3); however, more than 75% of the oils were recovered in an aqueous medium. Additionally, the use of an aqueous solvent limits several drawbacks, specifically the high cost, but also hazards due to the volatility and flammability of organic solvents.

### 3.6. UEAE Extracts Characterization

The raspberry, strawberry and blackberry pomace UEAE extracts were oil-in-water emulsions. They were lyophilized and then fully characterized. All results can be found in Table 4. The raspberry UEAE extract was constituted of 24 ± 2 g of lipidic compounds/100 g of dry extract (DE), while hydrophilic compounds accounted for 61 ± 7 g/100 g DE, with moisture assessed at 10 ± 5 g/100 g DE. Sugars and hydrolyzed proteins represented a major part of the hydrophilic compounds, with contents assessed at 19 ± 3 and 18 ± 0.4 g/100 g DE, respectively, followed by 13 ± 2 g of ashes/100 g DE. TPC was evaluated at 10.4 ± 0.9 g/100 g DE. Compared to our previous study [14], where a thermostable alkaline protease was used alone to recover simultaneously the hydrophilic and the lipophilic compounds from a raspberry PPC, the UEAE results were from the same order of magnitude, except for the lipidic content, which was nearly 3-fold higher in this study (24 ± 2 vs. 8.9 ± 0.4 g/100 g DE). This difference results from, on one hand, the coupling of the thermostable alkaline protease to US intensified the lipidic compounds extraction rate and, on the other hand, the raspberry PPC contained 3 times less oil compared to the raspberry pomace used in the present study (16 ± 0.3 g/100 g DP vs. 5.7 ± 0.2 g/100 g PPC). Overall, the strawberry and blackberry UEAE extracts displayed the same composition, except for the sugar contents, which were about 40 g/100 g DE (Table 4) and the lipidic contents, which were assessed at 3.5 ± 0.4 and 14 ± 0.2 g/100 g DE, respectively. Juśkiewicz et al. (2015) reported the same phenolic content for an aqueous polyphenolic extract obtained from a seedless strawberry PPC by EAE with Viscozyme L, which was evaluated to 7.8% of the total extract composition. The rest of the extract proximate composition varies, with protein and ash contents twice lower (8.0 and 7.5%, respectively) as obtained in this study. These differences may find their sources in the nature of the vegetal matrix, as this study valorized a strawberry pomace containing a mix of achenes and seeds, while Juśkiewicz et al. worked on a seedless strawberry PPC [43].

The UEAE allowed for the recovery of twice as much material from the pomaces compared to the organic solvent extraction used in comparison: 38 ± 0.2 vs. 16 ± 0.4 g/100 g DE for the raspberry pomace extracts; 42 ± 0.3 vs. 21 ± 0.3 g/100 g DE for the strawberry pomace extracts; and 51 ± 2 vs. 26 ± 0.3 g/100 g DE for the blackberry pomace extracts. These differences may come from the fact that UEAE was based on the vegetal matrix destruction (hydrolytic effect of the enzyme and cavitation phenomenon of US), while the extraction with the organic solvent (methanol:acetone:water, 7:7:6, *v*/*v*/*v*) was based on the diffusion of the compounds of interest from the vegetal matrix to the solvent. As the vegetal matrix was not degraded, less material was consequently extracted due to a limited mass transfer rate.

Lipophilic compounds (fatty acids, phytosterols and tocols) were extracted from each UEAE extract and then compared to the composition of their respective pomace oil obtained by conventional extraction with hexane (Table S1, Supplementary Materials). A high level of PUFAs was found in the raspberry UEAE oil (85% of the total fatty acid content, with 53 ± 0.5% of C18:2, n-6 and 32 ± 0.6% of C18:3, n-3), while saturated fatty acids were only present in low proportion (4 ± 2%). Omega-6 and omega-3 are classified as essential fatty acids, as they are mandatory to humans but not synthesized by the body, so they consequently have to be provided by food [44]. Moreover, the omega-6/omega-3 ratio assessed at 1.7 ± 0.1 is interesting, as the recommended ratio for health benefits stands between 1 and 5 [35]. A diet with excessive omega-6 intakes and poor omega-3 intakes could indeed lead to cardiovascular, auto-immune, inflammatory and neuropathologic diseases [45]. This fatty acid profile and omega-6/omega-3 ratio are consistent with our previous study [14] and with the work of Teng and al. (2016), where oil was extracted from raspberry seeds in ethanol with an ultrasonic bath for 37 min at 54 °C [11]. Additionally, the raspberry UEAE oil was also rich in phytosterols (927 ± 133 mg/100 g of oil), β-sitosterol being the dominant component found, accounting for 98% of the total phytosterol content. Phytosterols have numerous benefits to human health, as they are anti-inflammatory, anticancer, antioxidant and more. Their main benefit is their anti-arteriosclerotic effect, as a daily phytosterol intake could reduce blood cholesterol levels by limiting cholesterol absorption through competition [46]. High amounts of tocols were also found (222 ± 16 mg/100 g of oil) in the raspberry UEAE oil, with 75 ± 4% of γ-tocopherol and 18 ± 4% of α-tocopherol. Tocols show prominent antioxidant activities as well as a structural role, as they stabilize cell membranes [8]. The contents and profiles of phytosterols and tocols recovered in the UEAE oil are like the ones from the oil obtained with hexane. These results are coherent with our previous study, where the phytosterol and tocol amounts recovered in the enzymatically extracted oil were similar to the ones obtained for the oil extracted by hexane [14]. The tocol amount reported by Teng et al. (2016) from a raspberry oil was 7 times higher (15.21 ± 0.59 mg/g dry weight) [11], a difference that may come from the analytic method used to quantify the tocol contents (spectrophotometric method with α-tocopherol as standard for Teng et al. vs. HPLC method with a mixture of tocopherols and tocotrienols as standards in this study). For their part, the blackberry and strawberry UEAE extracted oils exhibit almost the same composition profile as the one obtained for the raspberry UEAE oil, as shown in Table S1 (Supplementary Materials). Their results were also similar to the oils obtained with hexane (Table S1). Blackberry UEAE oil fraction stood out for its interesting omega-6/omega-3 ratio (4.2 ± 0.1). The blackberry and strawberry UEAE oil fatty acids, phytosterols, and tocols contents and profiles determined in this study are similar to those found for cold-pressed oils obtained from blackberry and strawberry seeds [5,47]. Furthermore, as the composition of the oils extracted from the raspberry, strawberry and blackberry UEAE extracts are similar to the composition of the oils obtained from the respective pomaces by hexane at RT, the UEAE preserved the quality of the oils extracted from the three pomaces.

The antioxidant capacities of the three extracts were assessed by DPPH^●^ radical-scavenging assay (Table 4). The active polyphenol content of the raspberry UEAE extract was assessed at 3.9 ± 0.09 g/100 g DE, corresponding to an antioxidant capacity of 38 ± 2% (expressed as the ratio between active and total polyphenol contents). This antioxidant capacity is similar to the one displayed by the extract obtained with a conventional extraction using methanol:acetone:water (7:7:6, *v*/*v*/*v*) as solvent (Table 1). This is similar to the results of our previous work [14]. For their part, the strawberry and blackberry UEAE extracts showed the same trend, with antioxidant activities assessed, respectively, at 37 ± 0.2% and 48 ± 2%. The ET contents were also determined after total acidic hydrolysis. ETs were the main polyphenols found in the extracts, representing 26 ± 2%, 25 ± 2% and 40 ± 3% of the raspberry, strawberry and blackberry UEAE total polyphenols, respectively (Table 4), which is similar to the results of our previous work [14]. However, different results were found by Juśkiewicz et al. (2015) for a seedless strawberry PPC extract obtained by EAE (90 min at 65 °C with Viscozyme L), where 96% of the TPC was ETs.

### 3.7. UEAE Phenolics Identification by UPLC-DQD-ESI-MS/MS

Polyphenolic compounds present in each UEAE extract and their respective organic solvent extracts (methanol:acetone:water solvent, 7:7:6, *v*/*v*/*v*) were identified by UPLC-MS/MS. All results are summarized in Table 5.

According to the UV profile (Figure S1, Supplementary Materials) and mass data (ions and their fragments) of raspberry pomace extracts (organic solvent extraction vs. UEAE), the same phenolic compounds were identified, mostly ellagitannins (ETs) and their derivatives. Two characteristic ETs were identified at different retention times (*Rt*)—sanguiin H-6 or isomer (*Rt* 11.62 min) and lambertianin C (*Rt* 11.43 min)—which are, respectively, dimer and trimer of dihexahydoxydiphenoyl-galloylglucose (di-HHDP-GG). In both extracts, sanguiin H-6 concentrations were more important than those of lambertianin C. According to area proportions (aera of identified peak per total area of identified peaks, %), sanguiin H-6 represented a concentration of 42.7% and 32.2% of the organic solvent and UEAE-identified phenolics, respectively. Another form of sanguiin H-6 isomer, at a lower concentration, was also identified at a different retention time (*Rt* 10.37 min), with slightly different proportions in both extracts (11.3% vs. 8.5% in conventional and UEAE extracts, respectively). The concentration of lambertianin C was higher in the conventional extract, i.e., 11.7%, while it was only 4.5% in the UEAE extract. Overall, these two characteristic raspberry ETs (sanguiin H-6 or isomer and lambertianin C) together constituted up to 66% and 45%, respectively, of the total content of identified phenolics of the conventional and UEAE extracts. Such results are supported by different authors [48,49,50]. These two compounds are the dominant ETs present in berries, especially blackberry, raspberry and strawberry. Other minor ET components were identified: galloyl-di-HHDP-glucose at *Rt* of 9.86 min (casuarictin or isomer), which is a characteristic monomer frequently found as constituents of the oligomeric ETs in *Rubus*, two sanguiin H-10 isomer, roshenin B (degalloylated sanguiin H-6) and other ETs derivatives, such as tellimagrandin I. Similar results have been reported in the literature [49,51,52]. However, a decrease can be noted for the proportion of sanguiin H-6 and lambertianin C in the UEAE extract in favor of the apparition of HHDP derivatives, such as glucose-HHDP-galloylglucose (GG) and more free ellagic acid and their conjugates (ellagic acid pentoside, methyl ellagic acid). The concentration of free ellagic acid (*Rt* 11.97 min) was significantly increased from 2.4% in the conventional extract to 17.5% in the UEAE extract. Although the presence of free ellagic acid and its conjugates has already been reported in the literature in different raspberry cultivars [49], these observations can be explained by the conditions applied during the UEAE process (pH 8, 60 °C and 20% US amplitude). These conditions can indeed induce partial degradation of the dimeric or trimeric ETs (sanguiin H-6 and lambertianin C) into smaller ET components. These results are in correlation with our previous work [14] and the ascertainment of Daniel et al. (1991), on the effect of alkaline pH in partial hydrolysis of crude ETs [53]. Other authors have reported the effect of temperature (>60 °C) and neutral or even slightly alkaline pH on the degradation of lambertianin C and sanguiin H-6 into intermediate products, such as sanguiin H-10 and galloyl-HHDP glucose [54]. Besides the major groups of hydrolysable tannins (lambertianin C and sanguiin H-6), a small amount of condensed tannins was identified in both extracts (conventional and UEAE), whose concentration varied slightly from one extract to another (Table 5). These were monomeric catechin, (epi)cat-epiafzelechin, and dimeric procyanidin. Such compounds were already reported for *Rubus* species [50,55,56].

As for raspberry pomaces, ETs represent a major part of the identified phenolics of both strawberry extracts (organic solvent vs. UEAE), as reported in Table 5. Agrimoniin (*Rt* 13.02 min, dimer of di-HHDP-GG) was the main ET found for both extracts, with proportions of 35.8% and 13.1%, respectively, of the total identified phenolics in organic solvent and UEAE extracts. Agrimoniin is known to be the principal ET of strawberries [57]. Lambertianin C isomer (*Rt* 13.58 min) was the second oligomeric ET identified, especially in the organic solvent extract, but was almost at trace level in the UEAE extract (Table 5). As for the previous one, a second lambertianin C derivative (*Rt* 12.34 min) was found with the same difference in proportion for each extract. It is a trimer of di-HHDP-GG minus an HHDP group. The identification of these strawberry polyphenols is in agreement with the literature [58]. Moreover, the same trend can be noted for the HHDP units, such as di-HHDP-glucose (*Rt* 2.1 min), which is commonly known as pedunculagin, diHHDP-galloylglucose (casuarctin isomer) at a retention time of 9.91 min and its derivative with an ellagic acid (*Rt* 10.62 min). This is in agreement with the increase in the level of free ellagic acid (*Rt* 11.94 min), which was twice as high in the UEAE extract (39.5% compared to 15.9% of the total identified phenolics of UEAE and conventional extracts, respectively). As for the raspberry, these findings can be attributed to the partial hydrolysis of the oligomeric ETs under the effect of the alkaline pH and/or temperature of 60 °C, associated with US at an amplitude of 70% applied during the process. Additionally, another probable effect of pH is the appearance of brevifolin carboxylic acid, not described to date in strawberries, but which had been characterized by Tanaka et al. (1990) as a degradation product of HHDP esters under the effect of basic pH [59]. Finally, some flavonols were also identified, at lower concentrations, in the two strawberry pomace extracts. They were mainly kaempferol and its glycoside conjugate or its esters of hydroxycinnamic acids, especially of p-coumaric acid kaempferol glucoside (*Rt* 13.73 min) and kaempferol 3-O-coumaroylglucoside (*Rt* 15.86 min), which were previously reported in strawberry [60].

Blackberry polyphenols are characteristic of *Rubus* fruits. They are mainly of ellagitannin and derivative types, as is the case for raspberry and strawberry. The UPLC-MS/MS analysis of the blackberry extracts (organic solvent and UEAE extracts) confirmed these findings and identified thirteen ETs. The two most abundant ETs were identified as sanguiin H-6 and lambertianin C (Table 5). The sanguiin H-6 (eluted at *Rt* 11.62 min) is in higher proportion in each extract. Its proportion was more important in the organic solvent extract (44.2% against 29% in the UEAE extract). Regarding lambertianin C (*Rt* 11.41 min), it was three times more concentrated in the organic solvent extract (10.1% compared to 3.5% of the total identified phenolics of conventional and UEAE extracts, respectively). These results corroborate with those previously reported in blackberry ETs [52,61,62]. As for raspberry extracts, the 3rd oligomeric ET was identified as an isomer of sanguiin H-10 (*Rt* 6.57 and 10.57 min). Another minor ET has been identified as galloyl-di-HHDP galloylglucose or sanguiin H-2, eluted at respective retention times of 11.1 min and 13.82 min (peaks n° 6 and 12). These compounds have not previously been described in blackberry but were identified in boysenberry [52]. Sanguiin H-2 has been reported in *Rubus* leaves and as a minor hot-water degradation product of lambertianin C by Tanaka et al. (1993) [63]. In our previous work, sanguiin H-2 was also identified in the polyphenolic extract of raspberry PPC and as a consequence of the extraction conditions (60 °C, pH 9) applied during the aqueous enzyme-assisted extraction process [14]. So, the presence of sanguiin H-2 in UEAE blackberry extract may thus be an artifact of the extraction conditions applied during the UEAE process (pH 8, 60 °C and 70% US amplitude). In addition to these multimeric forms of ETs, primary units, such as di-HHDP glucose or pedunculagin (peak n°1, *Rt* 2.07 min) and a pedunculagin isomer (peak n°4, *Rt* 4.70 min) and their galloylated form casuarctin isomer (*Rt* 12.34 min) were found. These compounds are widely described in the literature in the *Rosaceae* family, in particular in *Rubus* fruits (especially for strawberry, boysenberry, blackberry) [52,58,62]. Another very notable finding in the UEAE extract was the high level of free ellagic acid (*Rt* 11.9 min), for which the proportion has reached 27%, against only 8% in the solvent organic extract.

Overall, the three UEAE extracts (raspberry, strawberry and blackberry) had a high proportion of ellagic acid, due probably to the partial hydrolysis of the oligomeric ETs under the effect of the alkaline pH and/or temperature of 60 °C, associated with US amplitude applied during the extraction process.

The state of the art on ETs and ellagic acid conjugates has been reported in many works focused on the potential health-related activities of these compounds. These are mainly anti-inflammatory, antimicrobial and anticancer, and they reduce gastric ulcers [64,65,66,67]. In addition, ETs are reported to have strong antioxidant properties, which is attributed to the large number of hydroxyl groups present in their structure [50,68,69]. Compared to conventional extraction using organic solvents, the UEAE process induced partial hydrolysis of the oligomeric ETs in the three studied pomaces (raspberry, strawberry and blackberry). Overall, the extraction conditions may have an impact on the structural integrity of the sanguiin H-6 and lambertianin C, but the compounds that appeared, in particular sanguiin H-10, all the HHDP sub-conjugates and especially free ellagic acid, remain, as them, compounds of biological interest. Previous works have indeed supported their biological activities [70,71]. In general, the ultimate fate of ETs in the gastrointestinal tract is their complete degradation into free ellagic acid. ET metabolism indeed takes place in the small intestine where the physiological pH causes their hydrolysis and release into ellagic acid, which will, in turn, be metabolized by the intestinal microflora into several urolithin metabolites and then will be absorbed and found in plasma [72,73].

## 4. Conclusions

This study demonstrated the feasibility of combining a thermostable alkaline protease with ultrasounds (US) to obtain, in one single step, berry pomace extracts that are rich in both lipophilic and hydrophilic compounds. First, a thermostable alkaline protease was selected amongst another acid protease and a mix of carbohydrolases for its ability to extract, in an aqueous medium, significant amounts of polyphenol and oil in a short period. Next, the simultaneous combination of this enzyme to US was chosen for optimization amongst two other sequential combinations, as it showed the best compromise between polyphenol and oil extraction rates, easiness of implementation and conceptual break with existing literature. The ultrasound-enzyme-assisted extraction (UEAE) was optimized by an experimental definitive screening design (DSD), and optimum parameters were set at: US amplitude = 20% for the raspberry pomace and 70% for the strawberry and blackberry pomaces; pH = 8; E/S ratio = 1% (*w*/*w*); S/L ratio = 6% (*w*/*v*); extraction time = 30 min; T = 60 °C. In these conditions, more than 100% of the total polyphenols and up to 75% of the oil were simultaneously recovered from the pomaces, in an aqueous medium. More precisely, 4.9 ± 0.1 g of total polyphenols, 1.6 ± 0.02 g of active polyphenols and 12 ± 0.05 g of oil per 100 g of raspberry dry pomace (DP) were extracted (efficiencies of, respectively, 104 ± 3%, 76 ± 0.5% and 76 ± 0.8% compared to organic solvent extractions used as references). For the strawberry pomace, 4.4 ± 0.09 g of total polyphenols, 1.6 ± 0.06 g of active polyphenols and 3.5 ± 0.2 g of oil per 100 g DP were recovered (respectively, 117 ± 3%; 84 ± 3% and 55 ± 3% of efficiency). Regarding the blackberry pomace, yields were 5.3 ± 0.2 g of total polyphenols, 2.2 ± 0.03 g of active polyphenols and 11 ± 0.1 g of oil per 100 g DP (efficiencies of 105 ± 4%; 78 ± 1% and 64 ± 0.6%, respectively). The UEAE was compared, for the three pomaces, with single enzyme-assisted extraction (EAE) and ultrasound-assisted extraction (UEA) (realized under the same extraction parameters), to assess the synergy between the enzyme and US. For the raspberry and strawberry pomaces, the coupling displayed a protective effect towards the polyphenol antioxidant activity and boosted the oil extraction rate, while for the blackberry pomace, combining the enzyme to US showed an additive synergistic effect towards the oil extraction rate.

The UEAE extracts were oil-in-water emulsions, rich in PUFAs, tocols, phytosterols and ellagitannins. Their compositions were similar to phenolic and lipidic fractions obtained from their respective pomace with conventional extractions (performed at RT), using, respectively, methanol:acetone:water (7:7:6, *v*/*v*/*v*) and hexane as solvents, which means that the UEAE process preserved the quality of the extracted bioactive compounds. Considering the versatility of the bioactive compounds recovered, the UEAE extracts may be interesting for the nutraceutical field.

## Figures and Tables

**Table 1:** Chemical composition of the raspberry, strawberry and blackberry pomaces.

Pomace	Raspberry	Strawberry	Blackberry
Moisture	4.7	6.4	4.9
Ashes	1.4 ± 0.07	2.9 ± 0.6	2.3 ± 0.2
Fat	16 ± 0.3	6.3 ± 0.1	18 ± 0.3
Proteins	8 ± 0.4	10 ± 2	9.5 ± 0.05
Total fibers	80 ± 3	74 ± 0.1	62 ± 2
Fibers profile (% of total fibers)			
Pectin	0.4 ± 0.02	8 ± 1	4.1 ± 0.1
Soluble lignin	0.3 ± 0.1	0.4 ± 0.01	0.4 ± 0.06
Klason lignin	34 ± 0.4	28 ± 2	28 ± 0.3
Hemicellulose	6 ± 0.3	9 ± 0.6	7 ± 0.5
Cellulose	79 ± 2	57 ± 3	67 ± 2
TPC	4.7 ± 0.2	3.8 ± 0.1	5.1 ± 0.1
Ellagitannins ^1^	1.4 ± 0.05	1.5 ± 0.2	2.9 ± 0.3
Anthocyanins ^2^	10 ± 2	27 ± 9	99 ± 11
Active polyphenol content ^3^	2.0 ± 0.07	1.9 ± 0.04	2.9 ± 0.2

Mean values are expressed with standard deviations (*n* = 3). DP: dry pomace; TPC: total phenolic content. ^1^: Ellagitannin content determined as total ellagic acid equivalent after HCl hydrolysis in methanol. ^2^: Expressed in mg/100 g of DP. ^3^: Active polyphenol content measured by DPPH^●^ radical-scavenging assay. Results are expressed as g/100 g of DP and the anthocyanin content is expressed in mg/100 g of DP.

**Table 2:** DSD experimental design for the optimization of UEAE process on raspberry pomace, including analytical factors and levels for DSD model.

Independent Factors							Levels		
							−1	0	1
U_1_	US amplitude (%)						20	45	70
U_2_	pH						7	8	9
U_3_	E/S ratio (%, *w*/*w*)						1	2	3
U_4_	S/L ratio (%, *w*/*v*)						3	6	9
U_5_	Extraction time (min)						30	45	60
U_6_	Temperature (°C)						40	50	60
							Extraction yields (g/100 g DP)		
Assay	U_1_	U_2_	U_3_	U_4_	U_5_	U_6_	Total polyphenols	Active polyphenols	Oil
1	0	1	1	1	1	1	4.9 ± 0.30	1.6 ± 0.02	11.7 ± 0.02
2	0	−1	−1	−1	−1	−1	2.2 ± 0.20	0.9 ± 0.08	10.0 ± 0.01
3	1	0	1	−1	−1	1	4.4 ± 0.20	1.7 ± 0.05	13.2 ± 0.20
4	−1	0	−1	1	1	−1	3.2 ± 0.02	1.1 ± 0.07	11.1 ± 0.01
5	1	1	0	1	−1	−1	4.3 ± 0.30	1.2 ± 0.30	11.9 ± 0.20
6	−1	−1	0	−1	1	1	3.6 ± 0.20	1.5 ± 0.04	12.5 ± 0.60
7	1	−1	1	0	1	−1	1.9 ± 0.09	0.9 ± 0.02	8.6 ± 0.30
8	−1	1	−1	0	−1	1	4.5 ± 0.10	1.6 ± 0.06	12.3 ± 0.20
9	1	−1	−1	1	0	1	2.3 ± 0.06	1.1 ± 0.08	9.5 ± 0.20
10	−1	1	1	−1	0	−1	4.2 ± 0.10	1.4 ± 0.05	12.6 ± 0.20
11	1	1	−1	−1	1	0	4.2 ± 0.20	1.4 ± 0.05	12.8 ± 0.06
12	−1	−1	1	1	−1	0	2.6 ± 0.20	0.9 ± 0.02	9.0 ± 0.20
13	0	0	0	0	0	0	3.9 ± 0.04	1.4 ± 0.04	11.9 ± 0.10
14 ^1^	−1	0	−1	0	−1	1	4.9 ± 0.10	1.6 ± 0.02	12.0 ± 0.05
Conventional extractions ^2^	-	-	-	-	-	-	4.7 ± 0.20	2.1 ± 0.04	15.7 ± 0.30

Mean values with standard deviations (*n* = 3). ^1^: Experiment 14 is a combination of experiments 3 and 8 and was designated as the optimized extraction. ^2^: Extraction in methanol:acetone:water (7:7:6, *v*/*v*/*v*) for total and active polyphenols recoveries and in hexane for oil recovery, designated as references.

**Table 3:** Extraction performances from the UEAE vs. conventional extractions.

Pomace	Raspberry		Strawberry		Blackberry	
Extract	Conventional Extractions ^1^	UEAE	Conventional Extractions ^1^	UEAE	Conventional Extractions ^1^	UEAE
Sugars	5.8 ± 0.8 ^a^	7.2 ± 0.9 ^a^	9 ± 1 ^b^	15.3 ± 0.5 ^a^	14 ± 1 ^b^	22 ± 3 ^a^
Proteins	-	7.0 ± 0.2	-	7.0 ± 0.6 ^a^	-	7.5 ± 0.9 ^a^
Lipids	16 ± 0.3 ^b^	12 ± 0.1 ^a^	6.3 ± 0.1 ^b^	3.5 ± 0.2 ^a^	18 ± 0.3 ^b^	11.2 ± 0.1 ^a^
TPC	4.7 ± 0.2 ^a^	4.9 ± 0.1 ^a^	3.8 ± 0.1 ^b^	4.40 ± 0.1 ^a^	5.1 ± 0.1 ^a^	5.3 ± 0.2 ^a^
Ellagitannins ^2^	1.4 ± 0.05 ^b^	1.04 ± 0.03 ^a^	1.5 ± 0.2 ^b^	1.0 ± 0.2 ^a^	2.9 ± 0.3 ^b^	2.07 ± 0.09 ^a^
Active polyphenol content ^3^	2.0 ± 0.07 ^b^	1.5 ± 0.04 ^a^	1.9 ± 0.04 ^b^	1.6 ± 0.06 ^a^	2.9 ± 0.2 ^b^	2.2 ± 0.03 ^a^
Antioxidant activity ^4^ (%)	42 ± 2 ^b^	36 ± 2 ^a^	50 ± 3 ^b^	37 ± 0.2 ^a^	57 ± 1 ^b^	47 ± 2 ^a^

Mean values are expressed with standard deviations (*n* = 3). DP: dry pomace; TPC: total phenolic content. ^1^: Conventional extractions: hexane for oil recovery and methanol:acetone:water (7:7:6, *v*/*v*/*v*) for total and active polyphenols recoveries. ^2^: Ellagitannin content determined as total ellagic acid equivalent after HCl hydrolysis in methanol. ^3^: Active polyphenol content measured by DPPH^●^ radical-scavenging assay. ^4^: Calculated as the ratio between active polyphenol and total polyphenol contents. Results are expressed as g per 100 g of DP. ANOVA (*p* < 0.05): two different letters indicate significantly different results.

**Table 4:** Chemical composition of the raspberry, strawberry and blackberry UEAE extracts.

Composition (g/100 g)	UEAE Raspberry	UEAE Strawberry	UEAE Blackberry
Moisture	10 ± 5	16 ± 1	18 ± 1
Sugars	19 ± 3	37 ± 1	42 ± 5
Lipids	24 ± 2	3.5 ± 0.4	14 ± 0.1
Proteins	18 ± 0.4	17 ± 2	15 ± 2
Ashes	13 ± 2	14 ± 0.6	8 ± 2
TPC	10.4 ± 0.9	9.1 ± 0.5	10.1 ± 0.7
Ellagitannins ^1^	2.7 ± 0.1	2.3 ± 0.3	4.0 ± 0.3
Active polyphenol content ^2^	3.9 ± 0.09	3.4 ± 0.2	4.8 ± 0.2

Mean values are expressed with standard deviations (*n* = 3). DE: dry extract; TPC: total phenolic content. ^1^: Ellagitannin content determined as total ellagic acid equivalent after HCl hydrolysis in methanol. ^2^: Active polyphenol content measured by DPPH^●^ radical-scavenging assay. Results are expressed in g/100 g DE.

**Table 5:** UPLC-DAD-ESI-MS/MS analysis of polyphenolic compounds in raspberry, strawberry and blackberry pomaces’ UEAE and conventional extracts.

Peak No.	RT (min)	Pic Proportion (%) ^1^		*m*/*z* [M-H]^−^	Ions Fragments	Compounds Identification
		Raspberry Pomace				
		Conventional Extract ^2^	UEAE Extract			
1	4.70	0.4	1.7	[633.07]^−^	[300.99]^−^, [275.02]^−^	HHDP-galloylglucose (GG)
2	5.02	1.1	3.6	[633.07]^−^	[300.99]^−^, [275.02]^−^	HHDP-GG
3	5.63	0.7	1.8	[289.07]^−^	[245.08]^−^, [179.03]^−^	Catechin
4	6.35	1.4	1.6	[858.07]^2−^	[783.07]^−^, [633.07]^−^, [469.00]^−^, [314.98]^−^, [300.99]^−^, [275.02]^−^	Degalloylated sanguiin H-6 (Roshenin B)
5	6.49	3.5	3.5	[783.07]^2−^	[897.04]^−^, [633.07]^−^, [469.00]^−^, [331.07]^−^, [314.98]^−^, [300.99]^−^,	Sanguiin H-10 or isomer
6	6.52	4.8	1.5	[577.13]^−^	[407.08]^−^, [289.07]^−^, [245.08]^−^, [125.02]^−^	Procyanidin dimer
7	7.63	9.7	7.3	[561.14]^−^	[407.08]^−^, [289.07]^−^	(epi)cat-epiafzelechin
8	9.80	1.1	1.9	[783.07]^2−^	[897.04]^−^, [633.07]^−^, [314.98]^−^, [300.99]^−^, [275.02]^−^	Sanguiin H-10 or isomer
9	9.86	2.7	3.8	[935.08]^−^	[783.07]^−^, [633.07]^−^, [463.05]^−^, [300.99]^−^, [275.02]^−^,	Casuarictin or isomer
10	10.37	11.3	8.5	[934.07]^2−^	[300.99]^−^	Sanguiin H-6 or isomer
11	10.57	2.0	5.9	[783.07]^2−^	[633.07]^−^, [314.98]^−^, [300.99]^−^, [275.02]^−^	Sanguiin H-10 or isomer
12	11.43	11.9	4.5	[1401.61]^2−^	[897.04]^−^, [633.07]^−^, [469.00]^−^, [300.99]^−^	Lambertianin C
13	11.62	42.7	32.2	[934.07]^2−^	[633.07]^−^, [300.99]^−^	Sanguiin H-6 or isomer
14	11.84	0.6	0.5	[433.04]^−^	[300.99]^−^, [201.07]^−^	Ellagic acid pentoside
15	11.97	2.4	17.5	[300.99]^−^	-	Ellagic acid (EA)
16	13.61	0.9	0.2	[469.05]^2−^ [939.11]^−^	[617.08]^−^, [465.07]^−^, [295.05]^−^, [169.01]^−^	Pentagalloylglucose
17	13.83	1.2	1.5	[447.06]^−^	[315.01]^−^, [299.99]^−^	Methyl ellagic acid pentoside
18	14.31	0.9	2.3	[450.99]^−^	-	Ellagitannin derivative ^3^
19	14.64	0.8	0.2	[542.03]^2−^ [1085.08]^−^	[897.04]^−^, [745.03]^−^, [633.07]^−^, [450.99]^−^, [300.99]^−^	Ellagitannin derivative ^4^
		Strawberry pomace				
		Conventional extract ^2^	UEAE extract			
1	0.94	0.1	1.5	[169.01]^−^	[125.02]^−^	Gallic acid
2	1.18	1.4	3.1	[331.07]^−^	[169.01]^−^	Galloylglucose (GG)
3	2.10	2.5	4.2	[783.07]^−^	[300.99]^−^, [275.01]^−^	di-HHDP-Glucose or pedunculagin
4	6.65	1.5	22.4	[291.01]^−^	-	Brevifolin carboxylic acid
5	9.91	6.7	3.8	[935.08]^−^ [467.03]^2−^	[633.07]^−^, [463.05]^−^, [300.99]^−^, [275.01]^−^	di-HHDP-GG (casuarictin isomer)
6	10.62	10.7	2.6	[617.03]^2−^ [1235.07]^−^	[631.06]^−^, [469.00]^−^, [314.98]^−^, [300.99]^−^	di-HHDP-GG derivative with EA group
7	11.97	15.9	39.5	[300.99]^−^	-	Ellagic acid (EA)
8	12.34	5.2	1.3	[1250.10]^3−^	[935.08]^−^, [897.04]^−^, [633.07]^−^, [300.99]^−^	Lambertianin C derivative (trimer of di-HHDP-GG with loss of a HHDP group)
9	13.02	35.8	13.1	[934.07]^2−^	[935.08]^−^, [897.04]^−^, [783.07]^−^, [633.07]^−^, [613.05]^−^, [300.99]^−^	Agrimoniin
10	13.58	6.2	1	[1401.61]^2−^	[897.04]^−^, [783.07]^−^, [633.07]^−^, [450.99]^−^, [300.99]^−^	Lambertianin C isomer
11	13.73	2.2	4.4	[447.09]^−^	[285.04]^−^, [284.03]^−^	Kaempferol glucoside
12	14.00	3.0	0.8	[1009.07]^2−^	[897.04]^−^, [633.07]^−^, [450.99]^−^, [300.99]^−^	Ellagitannin (non identified)
13	15.86	8.8	2.3	[593.13]^−^	[447.09]^−^, [285.04]^−^, [284.03]^−^	Kaempferol 3-O-coumaroyl-glucoside
		Blackberry pomace				
		Conventional extract ^2^	UEAE extract			
1	2.07	5.5	2.7	[783.07]^−^	[783.07]^−^, [481.07]^−^, [300.99]^−^, [275.02]^−^	di-HHDP-glucose (pedunculagin)
2	4.70	3.8	1.5	[783.07]^−^	[783.07]^−^, [481.07]^−^, [300.99]^−^, [275.02]^−^,	di-HHDP-glucose (pedunculagin isomer)
3	4.97	2.0	11.5	[633.07]^−^	[300.99]^−^, [275.02]^−^	HHDP-GG
4	6.57	6.4	3.3	[783.07]^2−^	[633.07]^−^, [469.01]^−^, [314.98]^−^, [331.07]^−^, [300.99]^−^,	Sanguiin H-10 or isomer
5	10.56	3.8	9.4	[783.07]^2−^	[897.04]^−^, [633.07]^−^, [469.01]^−^, [314.98]^−^, [300.99]^−^	Sanguiin H-10 or isomer
6	11.10	1.5	4.8	[1103.08]^−^	[897.04]^−^, [745.03]^−^, [633.07]^−^, [469.00]^−^, [314.98]^−^, [300.99]^−^, [275.02]^−^	Galloyl-di-HHDP galloylglucose (ex. sanguiin H-2)
7	11.41	10.9	3.5	[1401.06]^2−^	[897.04]^−^, [633.07]^−^, [469.01]^−^, [314.98]^−^, [300.99]^−^	Lambertianin C
8	11.62	44.2	29.1	[934.07]^2−^	[897.04]^−^, [633.07]^−^, [469.07]^−^, [314.98]^−^, [300.99]^−^, [275.02]^−^	Sanguiin H-6 or Isomer
9	11.86	2.1	2.7	[433.04]^−^	[300.99]^−^	Ellagic acid pentoside
10	11.97	8.1	27.1	[300.99]^−^	-	Ellagic acid
11	12.34	5.7	0.9	[935.08]^−^	[633.07]^−^, [463.05]^−^, [300.99]^−^, [275.02]^−^	di-HHDP-GG (casuarictin isomer)
12	13.82	4.3	3.2	[1103.08]^−^ [551.04]^2−^	[935.08]^−^, [898.05]^−^, [633.07]^−^, [300.99]^−^, [275.02]^−^	Galloyl-di-HHDP galloylglucose (ex. sanguiin H-2)
13	14.63	1.8	0.2	[542.03]^2−^ [1085.08]^−^	[897.04]^−^, [745.03]^−^, [633.07]^−^, [450.99]^−^, [300.99]^−^, [275.02]^−^	Ellagitannin derivative ^3^

HHDP: Hexahydoxydiphenoyl; GG: Galloylglucose. ^1^: Area proportion (%) calculated as peak area per total area of identified peaks. ^2^: Methanol:acetone:water (7:7:6, *v*/*v*/*v*) extraction. ^3^: Gallic acid is substituted with ellagic acid with an intramolecular link, as valoneic trilactone, for example, or flavogallol. ^4^: Tellimagrandin I substituted with ellagic acid or gallic acid as in eucalbanin A, for example.

## Supplementary Materials

The following supporting information can be downloaded at: https://www.mdpi.com/article/10.3390/antiox12101793/s1, Figure S1: UV Chromatograms (280 nm) for UPLC-DAD-ESI-MS/MS identification of polyphenolic compounds in raspberry (A), strawberry (B) and blackberry (C) pomaces UEAE and conventional extracts. Peak numbers refer to the identified phenolic compounds (Table 5); Table S1: Composition of the lipidic fractions extracted from the raspberry, strawberry, and blackberry pomaces and UEAE extracts.
