# Supplementary material for: Optimized Single-Step Recovery of Lipophilic and Hydrophilic Compounds from Raspberry, Strawberry and Blackberry Pomaces Using a Simultaneous Ultrasound-Enzyme-Assisted Extraction (UEAE)
Source: Antioxidants (Basel). 2023 Sep 22;12(10):1793. doi: 10.3390/antiox12101793 (PMC10603877; doi:10.3390/antiox12101793)
Supplement: Supplementary file 1 [file antioxidants-12-01793-s001.zip › antioxidants-2542460-supplementary.pdf]

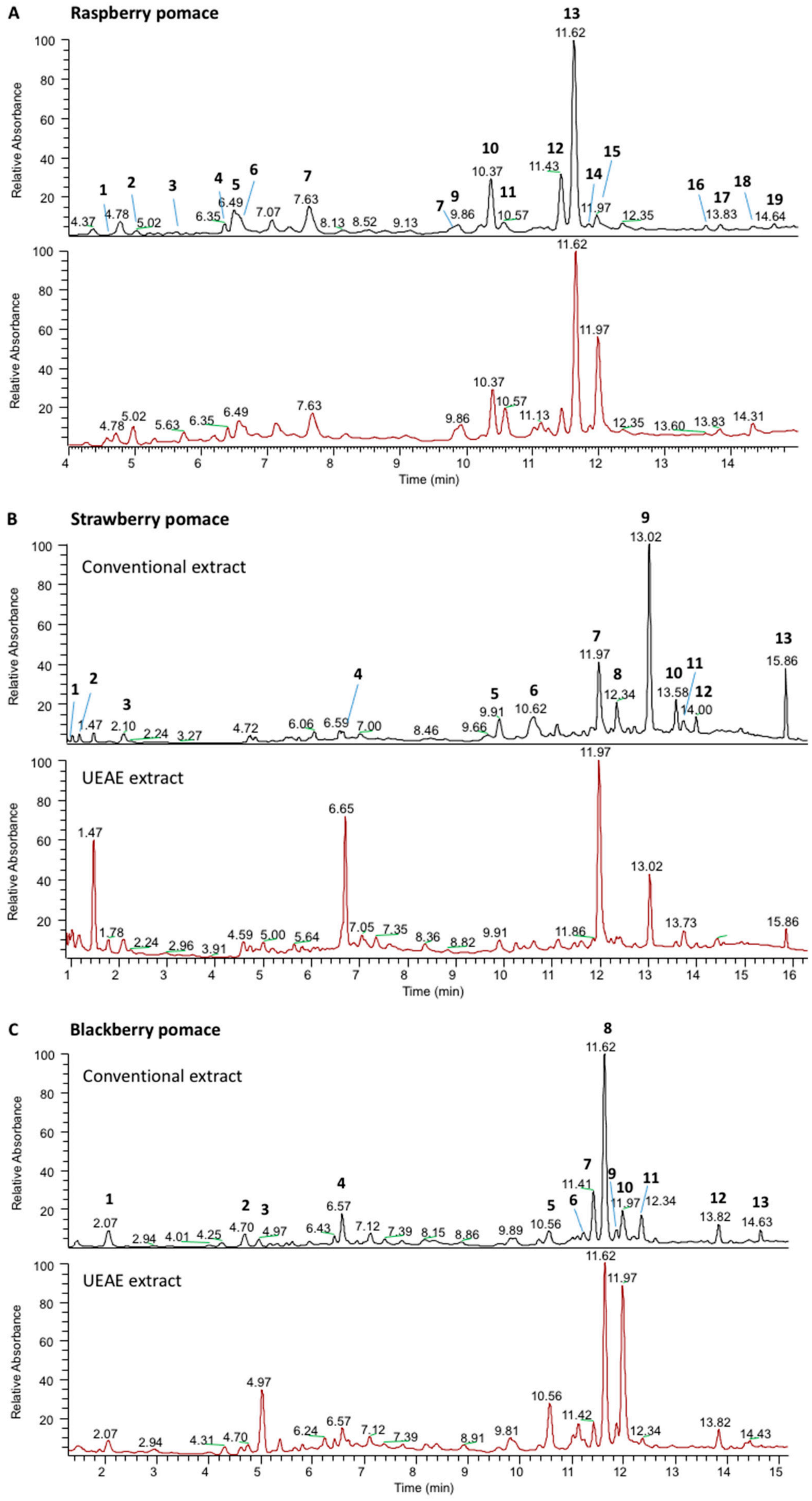
A
Raspberry pomace
Relative Absorbance
Time (min)
B
Strawberry pomace
Conventional extract
UEAE extract
Relative Absorbance
Time (min)
C
Blackberry pomace
Conventional extract
UEAE extract
Relative Absorbance
Time (min)

Figure S1: UV Chromatograms (280 nm) for UPLC-DAD-ESI-MS/MS identification of polyphenolic compounds in raspberry (A), strawberry (B) and blackberry (C) pomaces UEAE and conventional extracts. Peak numbers refer to the identified phenolic compounds (Table 5).

Table S1: Composition of the lipidic fractions extracted from the raspberry, strawberry, and blackberry pomaces and UEAE extracts.

	**Raspberry pomace**	**UEAE raspberry extract**	**Strawberry pomace**	**UEAE strawberry extract**	**Blackberry pomace**	**UEAE blackberry extract**
Fatty acid profile[1]						
C16:0	3.8 ± 0.8[a]	3 ± 1[a]	3.9 ± 0.6[a]	5.8 ± 0.2[b]	5.0 ± 0.2[a]	5.1 ± 0.3[a]
C18:0	1.6 ± 0.3[a]	1.1 ± 0.3[a]	1.2 ± 0.06[a]	2.0 ± 0.1[b]	4.3 ± 0.3[a]	4.3 ± 0.2[a]
C18:1	11 ± 2[a]	11 ± 0.1[a]	12 ± 0.9[a]	14 ± 0.4[b]	21 ± 2[a]	21 ± 0.9[a]
C18:2	52 ± 7[a]	53 ± 0.5[a]	46 ± 4[a]	45 ± 2[a]	55 ± 5[a]	55 ± 3[a]
C18:3	32 ± 5[a]	32 ± 0.6[a]	38 ± 4[a]	32 ± 0.8[b]	13 ± 2[a]	13 ± 0.5 [a]
C20:0	ND	ND	ND	1.3 ± 0.1	1.5 ± 0.04[a]	1.3 ± 0.05[a]
SFA	5 ± 1[a]	4 ± 2[a]	5.1 ± 0.6[a]	9.2 ± 0.3[b]	11 ± 0.5[a]	11 ± 0.5[a]
MUFA	11 ± 2[a]	11 ± 0.1[a]	12 ± 1[a]	14 ± 0.4[a]	21 ± 2[a]	21 ± 0.9[a]
PUFA	83 ± 11[a]	85 ± 2[a]	78 ± 8[a]	77 ± 2[a]	68 ± 6[a]	68 ± 3[a]
n-6/n-3 ratio	1.6 ± 0.2[a]	1.7 ± 0.1[a]	1.2 ± 0.2[a]	1.4 ± 0.1[b]	4.2 ± 0.1[a]	4.2 ± 0.1[a]
Total tocol content[2]	249 ± 8[a]	222 ± 16[a]	66 ± 5[a]	61 ± 5[a]	390 ± 20[a]	371 ± 10[a]
Tocol profile[1]						
α-tocopherol	17 ± 0.7[a]	18 ± 4[a]	38 ± 5[a]	39 ± 4[a]	14 ± 0.3[a]	16 ± 0.8[b]
γ-tocopherol	76 ± 3[a]	75 ± 4[a]	62 ± 3[a]	61 ± 5[a]	73 ± 4[a]	73 ± 3[a]
δ-tocopherol	7.3 ± 0.4[a]	7.1 ± 0.6[a]	NQ	NQ	13 ± 2[a]	12 ± 0.3[a]
Total phytosterol content[2]	1164 ± 7[a]	927 ± 133[a]	894 ± 23[a]	665 ± 69[b]	1074 ± 4[a]	771 ± 5[b]
Phytosterol profile[1]						
β-sitosterol	97 ± 0.8[a]	98 ± 14[a]	97 ± 3[a]	97 ± 2[a]	97 ± 0.5[a]	96 ± 0.5[a]
Campesterol	2.3 ± 0.3[a]	2.3 ± 0.3[a]	3.5 ± 0.4[a]	3 ± 0.2[a]	3.4 ± 0.2[a]	3.6 ± 0.2[a]
Stigmasterol	0.8 ± 0.1	NQ	NQ	NQ	NQ	NQ

Mean values are expressed with standard deviations (n = 3).

SFA: saturated fatty acids; MUFA: mono-unsaturated fatty acids; PUFA: poly-unsaturated fatty acids; ND: not detected; NQ: not quantified.

[1]: Expressed as a percentage of the total fatty acid or tocol or phytosterol content.

[2]: Expressed in mg of compounds per 100 g of oil.

Anova ($P < 0.05$): two different letters mean that the results are significantly different.
